# Spread of COVID-19 in urban neighbourhoods and slums of the developing world

**DOI:** 10.1098/rsif.2020.0599

**Published:** 2021-01-20

**Authors:** Anand Sahasranaman, Henrik Jeldtoft Jensen

**Affiliations:** 1 Division of Sciences and Division of Social Sciences, Krea University, Sri City, AP 517646, India; 2 Centre for Complexity Science and Department of Mathematics, Imperial College London, London SW72AZ, UK; 3 Institute of Innovative Research, Tokyo Institute of Technology, 4259, Nagatsuta-cho, Yokohama 226-8502, Japan

**Keywords:** epidemic, slums, COVID-19, urban, scaling, neighbourhood

## Abstract

We study the spread of COVID-19 across neighbourhoods of cities in the developing world and find that small numbers of neighbourhoods account for a majority of cases (*k*-index approx. 0.7). We also find that the countrywide distribution of cases across states/provinces in these nations also displays similar inequality, indicating self-similarity across scales. Neighbourhoods with slums are found to contain the highest density of cases across all cities under consideration, revealing that slums constitute the most at-risk urban locations in this epidemic. We present a stochastic network model to study the spread of a respiratory epidemic through physically proximate and accidental daily human contacts in a city, and simulate outcomes for a city with two kinds of neighbourhoods—slum and non-slum. The model reproduces observed empirical outcomes for a broad set of parameter values—reflecting the potential validity of these findings for epidemic spread in general, especially across cities of the developing world. We also find that distribution of cases becomes less unequal as the epidemic runs its course, and that both peak and cumulative caseloads are worse for slum neighbourhoods than non-slums at the end of an epidemic. Large slums in the developing world, therefore, contain the most vulnerable populations in an outbreak, and the continuing growth of metropolises in Asia and Africa presents significant challenges for future respiratory outbreaks from perspectives of public health and socioeconomic equity.

## 1. Introduction

In the wake of the novel coronavirus COVID-19 pandemic that is currently sweeping the planet, there is increasing concern over the impact on large urban slums in the developing world. This concern primarily stems from the nature of dwelling arrangements in developing cities, where large proportions of the population live in densely populated slums and shantytowns [1]. Broadly, slums are defined as ‘communities characterized by insecure residential status, poor structural quality of housing, overcrowding and inadequate access to safe water, sanitation and other infrastructure’ [2]. This definition emphasizes the fact that slums house the poorest and most vulnerable populations in cities. UN-Habitat estimated that approximately 30% of the urban population lived in slums in 2014, with significant geographical heterogeneity—the proportion was 55% for sub-Saharan Africa and 31% for southern Asia and 21% for Latin America [3]. The sheer scale of slums is further exacerbated by the density of the population in such settlements. Table 1 presents some statistics on the density of people living in some of the largest metropolises of the developing world and shows that these cities have high average population densities (and high slum populations), but individual slum neighbourhoods even within these cities often show population densities an order of magnitude higher, suggesting significant intra-city heterogeneity in densities of living.

**Table 1.** Densities in developing world metropolises and their slum neighbourhoods.

city	slum population (%)	average population density (per km^2^)	large slum	slum population density (per km^2^)
Mumbai, India	41	25 771	Dharavi	335 900
Cape Town, South Africa	35	1520	Khayelishta	10 120
Rio de Janeiro, Brazil	22	5231	Mare	30 400
Dhaka, Bangladesh	38	19 501	Korail	205 410
Lagos, Nigeria	70	18 788	Mushin	128 882
Manila, Philippines	31	44 866	Tondo	73 548

It is important to remember that the high population densities in developing cities are being attained without just building vertically (unlike cities like New York City, Seoul or Tokyo), with typical living conditions in slums described as small single room shacks (approx. 10 m^2^) with around five people living in them, situated adjacent to one another and with up to 10 families sharing a water tap and a pit latrine [4]. High population density achieved under such conditions, therefore, creates an environment rife for epidemic spread through air or water. Our specific concern relates to the spread of disease through such urban slums, which represent a critical feature of urbanization in developing nations [1], especially in the context of infectious disease outbreaks like COVID-19 where viral transmission is aided by increased population density, manifested as more frequent person-to-person contact, crowded housing and unsanitary environments [5,6].

The purpose of this work is twofold. First, we use COVID-19 caseload data at a sub-city level (ward or neighbourhood or local government level) to empirically characterize the spread of the epidemic across urban neighbourhoods in six developing world metropolises, specifically to understand the nature of infectious spread at fine-grained levels in contexts where slums are a salient feature of the urban landscape. Based on this characterization, we study the systematic differences in the spread of COVID-19 across slum and non-slum neighbourhoods in these cities. Second, we seek to create a network model of infectious spread through an urban system (city) to provide a candidate explanation of the empirically observed variation in caseloads across slums and non-slums. While there has been an emerging body of field-based studies and earth observations on COVID-19 in cities [7–10], our network modelling approach offers a new and different lens through which to explore the fine-grained spread of infection in urban neighbourhoods. We discuss the results obtained in the context of cities in the developing world.

## 2. Evidence on impact of COVID-19 on cities and slums

We focus our attention on six specific cities (table 1) because they are among the largest cities of the global south; are severely impacted by COVID-19; and have made available data at the required level of local granularity to enable this fine-grained analysis. However, even for many of these cities, data at the sub-city level is not released regularly and is only available occasionally. We discuss all sources of data and constraints in electronic supplementary material, appendix S1. Table 2 provides greater detail on the sub-city units we consider for the analysis.

**Table 2.** City and neighbourhood details.

city	nature of sub-city unit	number of sub-city units	average population of sub-city unit	caseload as of (date)	total caseload
Mumbai Corporation	ward	24	518 432	20 July 2020	99 566
City of Cape Town	suburb, township	58	64 483	22 June 2020	38 540
Rio de Janeiro	bairro	163	40 261	21 July 2020	61 818
Dhaka City	thana	41	161 711	28 June 2020	15 754
Lagos Metropolitan Area	local govt area (LGA)	16	1 375 405	7 May 2020	1352
City of Manila	district	16	110 429	10 July 2020	3248

**Figure 1.**
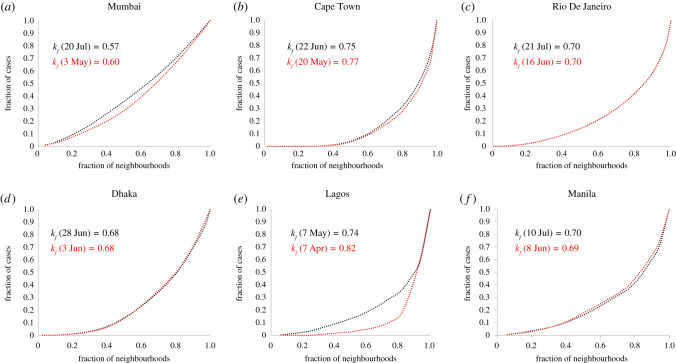
Distribution of COVID-19 cases across neighbourhoods. Fraction of cases versus fraction of neighbourhoods. (*a*) Mumbai. (*b*) Cape Town. (*c*) Rio de Janeiro. (*d*) Dhaka. (*e*) Lagos. (*f*) Manila. Black dots: most recent caseload distribution, dates as per table 2. Red dots: older caseload distributions—Mumbai (30 May), Cape Town (20 May), Rio de Janeiro (16 June), Dhaka (3 June), Lagos (7 April) and Manila (8 June). The *k*-index appears to decline over time.

We first study the distribution of cumulative caseloads across sub-city units (we will refer to these sub-city units generally as neighbourhoods) for each of the six cities and find that cases show an unequal distribution across neighbourhoods, with a high proportion of cases contained in a small proportion of neighbourhoods—the top 20% of neighbourhoods (ordered by COVID-19 caseload) account for 31% of cases in Mumbai, 69% in Cape Town, 58% in Rio de Janeiro, 50% in Dhaka, 65% in Lagos and 55% in Manila, respectively (figure 1, black). The emergence of such a relationship across neighbourhoods in all cities under consideration—given the underlying heterogeneity in terms of numbers of sub-city units, population scale of units and total caseload—suggests that the outcome is robust and representative of real underlying dynamics of infectious spread.

We characterize the unequal nature of this spread across neighbourhoods using the *k*-index, which is a measure of the inequality in the distribution of an attribute across a population [11]. In our context, we use the *k*-index as a measure of the inequality in distribution of COVID-19 cases across neighbourhoods in cities. The *k*-index is best understood as a metric that generalizes Pareto's 80–20 rule—an observation by Italian economist Vilfredo Pareto that approximately 80% of a nation's wealth belonged to only approximately 20% of the population [12]. Given the cumulative distribution of COVID-19 caseload across neighbourhoods, the *k*-index (*k_f_*) has the property that *k_f_* proportion of neighbourhoods contain (1 − *k_f_*) proportion of the cases and consequently the remaining (1 − *k_f_*) proportion of neighbourhoods account for *k_f_* proportion of the cases [11]. We find that, apart from Mumbai (*k_f_* = 0.57), all other cities have much higher *k_f_*—Cape Town, Rio de Janeiro, Dhaka, Lagos and Manila have *k_f_* = 0.75, 0.70, 0.68, 0.74, 0.70, respectively. This results in an average *k_f_* = 0.69 across all cities under consideration, meaning that while approximately 69% of the neighbourhoods in these cities account for only approximately 31% of reported cases, the remaining approximately 31% of neighbourhoods account for approximately 69% of cases. We also study the time evolution of the distribution of cases in these cities, considering two points in time that are around a month apart (subject to data availability as highlighted in electronic supplementary material, appendix S1), and find that the *k*-index of the distribution appears to decrease over time for most cities (Mumbai, Cape Town and Lagos), while it remains consistent for Rio de Janeiro and Dhaka, and marginally increases for Manila (figure 1, red).

When we explore the distribution of COVID-19 caseload across states or provinces within the countries containing these six cities, we find that average *k_f_* = 0.73, which is very similar to the *k*-index observed for caseload distribution within these cities. The distribution of cases across the states of India, states of Brazil, states of Nigeria, provinces of South Africa and districts of Bangladesh yield *k_f_* of 0.77, 0.65, 0.75, 0.70 and 0.76, respectively (we were unable to find province-level data for Philippines). Therefore, the distribution of caseload across states/provinces in nations mirrors the distribution across neighbourhoods in cities, indicating self-similar behaviour across scales.

Given this unequal distribution, we now explore the characteristics of neighbourhoods that have the highest caseloads. Our current understanding of COVID-19 suggests that physical proximity is an important determinant of local spread. Therefore, we study caseloads across neighbourhoods in all six cities, with a focus on differential impacts of COVID-19 on high-density neighbourhoods with slums, and other neighbourhoods.

In order to do this, we first map large slum settlements in these cities to the appropriate sub-city unit and label as ‘neighbourhoods with slums' only those sub-city units which show a high concentration of slums as revealed by slum mapping exercises (detailed in electronic supplementary material, appendix S2). It is important to point out that entire neighbourhoods are rarely classifiable as completely being slums or non-slums (and many neighbourhoods have both slum and non-slum components), but given the absence of slum population data at the fine-grained level of urban neighbourhoods, this separation into a slum/non-slum dichotomy allows for an approximate density-based characterization of urban neighbourhoods, and enables us to study the nature of the spread of the epidemic within cities.

The resulting list of neighbourhoods with slums across the six cities is: 11 out of 23 wards in Mumbai (G-North—containing the Dharavi slum, G-South, F-South, L, N, H-East, M-East, M-West, K-East, K-West, P-North); eight out of 58 suburbs/townships in Cape Town (Khayelitsha, Mitchells Plain, Gugulethu, Delft, Philippi, Nyanga, Langa, Mfuleni); 41 out of 163 bairros in Rio de Janeiro (Rocinha, Jacarezihno, Mare, Cidade de Deus, Complexo do Alemao, Mangueira, Penha, Acari, Tijuca, Costa Barros, Ramos, Benfica, Pavuna, Encantado, Lins de Vasconcelos, Manguinhos, Madureira, Inhaumos, Rio Comprido, Iraja, Anchieta, Vigario Geral, Guadalupe, Cordovil, Piedade, Jacare, Parada de Lucas, Copacabana, Tomas Coelho, Magalhaes Bastos, Realengo, Bangu, Jacarepagua, Andarai, Bras de Pina, Honorio Gurgel, Engenho Novo, Turiacu, Padre Miguel, Coelho Neto, Engenho de Dentro); 12 out of 41 thanas in Dhaka (Mirpur, Gulshan—containing the Korail slum, Mohammadpur, Jatrabari, Lalbagh, Sutrapur, Chak Bazar, Gendaria, Hazaribagh, Kotwali, Kamrangir Char, Shyampur); five out of 16 LGAs in Lagos (Agege, Ajeromi-Ifelodun, Mushin, Somolu, Lagos Island and Lagos Mainland—containing the floating Makoko slum); and two out of 17 districts in Manila (Tondo and San Andres).

**Figure 2.**
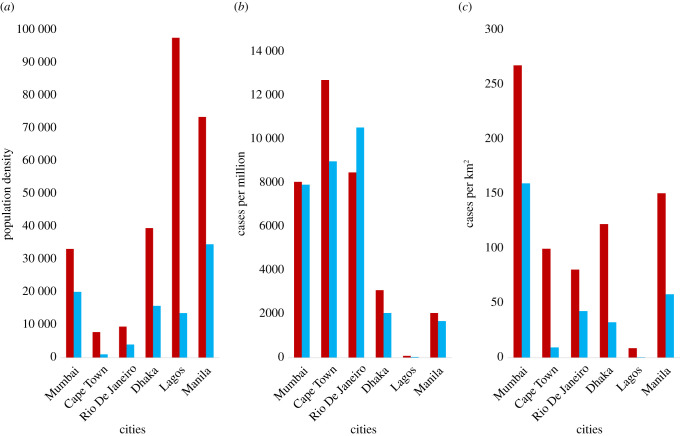
Distribution of COVID-19 cases across neighbourhoods with slums and other neighbourhoods. Red columns: neighbourhoods with slums. Blue columns: non-slum neighbourhoods. (*a*) Population density of slum and non-slum neighbourhoods shows that slum neighbourhoods have higher densities, on average, across all cities. (*b*) COVID-19 cases per million population across slum and non-slum neighbourhoods. Slum neighbourhoods are more affected in all cities, except Rio de Janeiro. (*c*) COVID-19 cases per square kilometre across slum and non-slum neighbourhoods. In all cities, slum neighbourhoods show much higher spatial density of cases than non-slum neighbourhoods.

We find, in line with expectations, that average population densities of neighbourhoods with slums are much higher than other neighbourhoods (figure 2*a*, neighbourhoods with slums—red, other neighbourhoods—blue). When we assess the distribution of cases across neighbourhood types taking into account population density, we find that caseload per capita represented by caseload per million population (figure 2*b*) and caseload per unit area (km^2^) (figure 2*c*) are systematically higher in neighbourhoods with slums than in non-slum neighbourhoods across cities.

The only exception here is Rio de Janeiro, where we find that neighbourhoods with slums have a lower caseload per capita than non-slum neighbourhoods; this should, however, be seen in light of the many concerns have been raised about testing and measurement of COVID-19 cases in Brazil's favelas [13–15]. A survey of Rio de Janeiro's favelas estimated that the number of people infected by COVID-19 in these slums could be 30 times official estimates, and that approximately 25% of those tested in the city's largest favela, Rocinha, were infected [16,17]. Even in the other cities in our analysis where cases per capita are higher in slums with neighbourhoods (as expected), there could be significant undercounting of caseloads in slums—for instance, a sero-survey across three wards in Mumbai found that approximately 57% of slum residents had developed antibodies to COVID-19 as compared to only approximately 16% non-slum residents [18]. Overall, this finding suggests that neighbourhood population density is a critical mediator of the dynamics of infectious spread in a city, and that the urban poor in slums are starkly worse off in terms of epidemy outcomes.

Given this empirical context, we present a computational model of the spread of a typical respiratory epidemic in a network representing a city-system composed of slum and non-slum neighbourhoods. Our objective is twofold: first, we seek to test whether the model provides a general explanation for the empirically observed systematic variance in infection caseloads across neighbourhoods (as in case of COVID-19); and second, to explore the evolution of cumulative and peak caseloads across neighbourhoods through the duration of the epidemic.

## 3. Model definition and specifications

We model a network of a city consisting of *N* nodes, with each node representing an agent in the city, and *H* neighbourhoods among which agents are distributed. While we lack empirical data on the structure of real networks of physical proximity in cities of the developing world, there is a growing body of work indicating that highly connected nodes or ‘super-spreaders’ are disproportionately important in the transmission of even influenza-like illnesses [19–22]. Population-level estimates of the basic reproduction number for an epidemic assume homogeneous populations, but it has been demonstrated that many epidemics are better described by heterogeneous transmission where certain individuals infect an unusually large numbers of secondary contacts (super-spreading events), while other individuals infect very few or none [21,22]. Emerging evidence from the COVID-19 pandemic suggests that super-spreading is a salient mechanism in the spread of this virus as well [23–25]. Therefore, we propose to explore the dynamics of transmission on a scale-free Barabási–Albert (BA) network [26]. The BA network with *N* nodes is generated by attaching new nodes with *m* neighbours, such that the links of a new node show preferential attachment for existing nodes with high degree. This results in a degree distribution where a few nodes have very high degrees, while many nodes have much lower degrees. We also test the robustness of model outcomes for sensitivity to network type in electronic supplementary material, appendix S3.

Our interest is in studying differential impacts across slum and non-slum neighbourhoods described by a wide variation in population density, and we simulate such density differences as differences in the average degree of nodes in each neighbourhood of the network. That is, we model connectedness of a neighbourhood as the average degree of nodes in a neighbourhood. We construct neighbourhoods in the network by ordering all *N* agents based on node degree and then allotting each of them in order to the *H* neighbourhoods in the city system, such that the first neighbourhood is filled with the first set of ordered *N*/*H* agents, followed by the second neighbourhood and so on, until the final neighbourhood is filled with the last set of *N*/*H* agents. Given the heterogeneity in neighbourhood evolution across cities, we do not assume a systematic positive relationship between neighbourhood populations and population densities—indeed, we find no evidence of a systematic relationship between population and density across the six cities under consideration.

In the absence of any reliable data on proximate daily contacts in the urban neighbourhoods of developing cities, creating neighbourhoods in this way ensures that average degree of nodes across neighbourhoods shows significant heterogeneity. It also means that nodes in neighbourhoods with higher average degree are connected to many nodes both within and outside of their neighbourhoods—this is meaningfully representative of the urban poor in cities of the developing world, who live in densely populated slums and have high interconnectedness (unavoidable physical proximity) within the slum, but work largely in other non-slum neighbourhoods, including as essential services workers such as sanitation and health workers. This algorithm also means that neighbourhoods with high average degree correspond only to high-density slum neighbourhoods, and not high-density neighbourhoods in general—for instance, neighbourhoods that are well-off and where high densities are obtained by building vertically are represented in our model as neighbourhoods with lower average node degree (lower connectedness), which is a more likely representation of their daily contact networks.

We use the three compartment susceptible–infected–recovered (SIR) model as the basis for an agent's progression through the duration of the epidemic [27]. Agents start out in the susceptible (*S*) compartment until the time they are infected, at which point they fall into the infected (*I*) compartment. After spending a specified duration of time being infected, when they spread the infection in the network, they move to the recovered (*R*) compartment, at which time they are immune—neither infective nor susceptible to the infection again. At *t* = 0 days, we have one random node that is infected (*I*), while the remaining *N* − 1 nodes are susceptible (*S*).

At each time step *t*, the dynamics of infectious spread in the network are modelled as follows: first, each infected (*I*) agent spreads the disease to each of its susceptible (*S*) neighbours in the network with transmission probability *p*. Given a node *i* with *q* neighbours (or a contact rate of *q*), the average daily infections caused by this node, or its daily transmission rate (*β_i_*), is the product of the transmission probability and the contact rate of the node, *β_i_* = *pq*. Second, each infected agent moves into the recovered (*R*) compartment if it has spent 1/*γ* days in the infected (*I*) compartment. *γ* is defined to be the recovery rate and remains constant through the dynamics. Like many other studies of COVID-19 [28–31], we use the canonical SIR model to explore dynamics of spread, but other variations such as the SEIR model (which includes an ‘exposed’ compartment containing individuals who have been infected but are not yet themselves infectious) have also been used to model the infection [32–34]. The qualitative nature of outcomes presented here would remain unchanged irrespective of the model chosen, though specific details such as timescales of the epidemy would change.

We propagate these dynamics over a period of *t* = *T_f_* days and study the distribution of cases across the *H* neighbourhoods over time, as well as the current and cumulative caseloads across neighbourhoods over time. Table 3 provides the complete set of parameter values and initial conditions for the simulations.

**Table 3.** Parameter values and initial conditions.

	values
*parameters*	
population—number of network nodes, *N*	10 000
number of edges from new node to extant nodes, m (for Barabási–Albert graph)	50
number of neighbourhoods, *H*	20
probability of node to node transmission, *p*	0.004
number of iterations (days) in one simulation of model *T_f_*	120
number of simulations	100
*initial conditions*	
number of susceptible nodes, *S*(0)	9999
number of infectious nodes, *I*(0)	1
number of recovered nodes, *R*(0)	0

As indicated earlier, the SIR dynamics are dependent on the transmission probability, which we simulate as *p* = 0.004 in the base case, and the contact density of nodes, which give the BA network structure, can show significant variation. For instance, a node with *q* = 50 connections, and with *γ* = 0.1, will produce an average of *pq*/*γ* = 2 infections. We also simulate the dynamics for *p* = 0.002 and *p* = 0.006 to study system outcomes for varying *p*. Similarly, while we choose *N* = 10 000 and *H* = 20 to define population and neighbourhoods in the base case, we also explore epidemy behaviour by varying system population across three orders of magnitude (*N* = 1000 and *N* = 100 000) as well as neighbourhood counts (*H* = 10 and *H* = 40), to ascertain whether observed outcomes are robust across system size specifications. Additionally, while the BA graph offers us a reasonable network structure with super-spreader nodes to mimic epidemic spread, we study the robustness of outcomes to network type by simulating dynamics on an Erdős–Rényi random graph [35]. Finally, we also vary the algorithm to populate neighbourhoods and create varying neighbourhood density profiles to ensure that model outcomes are not simply artefacts of the neighbourhood population mechanism used here. These varying densities could be seen as being reflective of decreasing contacts on account of non-pharmaceutical interventions such as physical distancing and lockdown. Detailed results for the various scenarios are presented in electronic supplementary material, appendix S3.

## 4. Results

**Figure 3.**
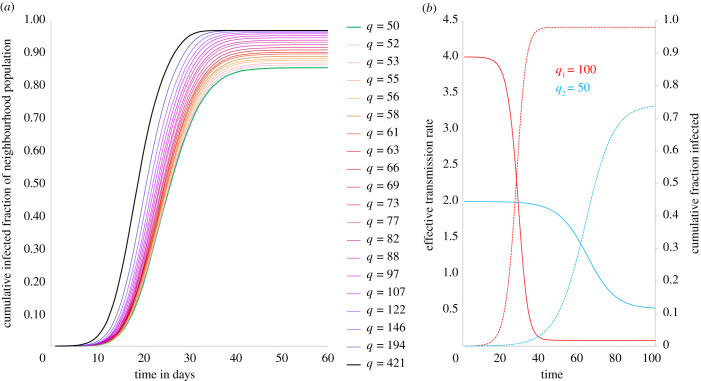
Cumulative caseload simulation and analytical description. (*a*) Simulation of cumulative infected population fractions across neighbourhoods over time. Higher density neighbourhoods not only show earlier and steeper rise in caseloads but also end up with higher fractions of populations infected at the end of the epidemic. (*b*) Effective transmission rate (and cumulative fraction infected) over time. Analytical description of the evolution of effective transmission rates for neighbourhoods with different average contact rates (*q*). Denser neighbourhood is represented by *q*_1_ = 100 (red) and sparser neighbourhood by *q*_2_ = 50 (blue).

The evolution of cumulative fraction of caseload across neighbourhoods clearly shows that the rate of case growth increases with population density (figure 3*a*). This is in keeping with the empirical finding that once epidemy dynamics are underway and the infection has reached higher density neighbourhoods, caseload per capita is higher in high-density neighbourhoods. For instance, at day 10 of the dynamics, the densest neighbourhood in our network (with average node degree, *q* = 421) has a cumulative caseload of 4.7% (as a fraction of its population), while the lowest density neighbourhood (with average node degree, *q* = 50) is at 0.5%, and all other neighbourhoods with densities in between these extremes show caseloads between 0.5% and 4.7% (figure 3*a*). The corresponding caseloads on days 20 and 30 are 66% and 96% for the densest neighbourhood, and 24% and 71% for the lowest density neighbourhoods.

To explore these dynamics analytically, consider a neighbourhood with *N_h_* nodes, each with degree *q*. Given *p* and *q*, at *t* = 0, the average daily transmission rate is *β* = *pq*. At the end of a time interval *t*, let *f_S_*(*t*) be the fraction of population still susceptible and *f_I_*(*t*) the fraction that has ever been infected until *t*, such that *f_S_*(*t*) = 1 − *f_I_*(*t*). *f_I_*(*t*) is given by (equation (4.1)):

4.1 $$f_{I}(t)=\{\begin{array}{ccc} \frac{1}{N_{h}}, & \text{at} & t=0\\ \;f_{I}(t-1)+(\beta f_{I}(t-1)(1-f_{I}(t-1)), & \;\text{for} & 1\leq t<1/\gamma \\ \;f_{I}(t-1)+(\beta \left(\;f_{I}(t-1)-f_{I}\left(t-\frac{1}{\gamma }\right)\right)(1-f_{I}(t-1)), & \;\text{for} & t\geq 1/\gamma \end{array}.$$

The effective transmission rate of the epidemic in the neighbourhood, *R_e_*(*t*), is the average number of people infected by an individual in the neighbourhood at time *t*:

4.2 $$R_{e}(t)=\frac{\;pf_{S}(t-1)q}{\gamma }=\beta f_{S}(t-1)/\gamma \;.$$

Using this simple construct, we consider two neighbourhoods—a slum with average degree *q*_1_ and a non-slum with average degree *q*_2_ (*q*_2_ < *q*_1_)—with probability of transmission *p* and a single node infected at *t* = 0. The evolution of *R_e_*(*t*) shows that the slum has a much higher effective transmission rate in the early part of the dynamics due to higher *q* (figure 3*b*). This results in sharp increase in caseloads in this period, causing a simultaneous sharp decline in *R_e_*(*t*) due to the coevolution of susceptible and infected populations. The non-slum neighbourhood has a lower effective transmission rate to begin with and shows a more gradual increase in cases. The overall effect is that higher density results in higher caseloads per capita in the slum as against the non-slum (figure 3*b*), which offers a possible explanation for the empirical observations from developing world cities where case density increases with neighbourhood population density (figure 2*b*,*c*).

**Figure 4.**
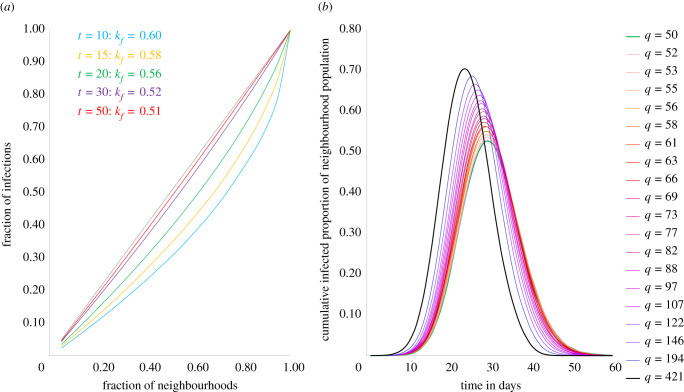
Distribution and peak caseload simulation. (*a*) Simulation of distribution of cumulative caseloads across neighbourhoods over time. Distribution of cases is more unequal earlier in the epidemic and become less unequal as epidemic progresses (*b*) Simulation of current caseloads across neighbourhoods over time. Higher density neighbourhoods show earlier and sharper peaks in active cases compared to lower density neighbourhoods. Dashed black line: line of equality (*y* = *x*).

We also study the distribution of cases across neighbourhoods and find that, just as observed empirically, there is an unequal distribution of caseload across neighbourhoods during the dynamics (figure 4*a*). However, as the epidemic runs its course, the inequality in distribution progressively reduces—figure 4*a* plots the distribution of caseloads at different points in time and we see that inequality in distribution of cases is greatest at *t* = 10 when *k_f_* = 0.62, following which there is continuous reduction in inequality until *t* = 50 when *k_f_* = 0.51, at which point the epidemic has ended. For the epidemic to end, it infects as much of the population as is required for the effective transmission rate to summarily decline below 1; therefore, even as dense slum neighbourhoods see their caseloads rise steeper and peak earlier (figure 4*b*), thus yielding higher inequality in case distribution, lower density non-slums are not immune to the epidemy and will see delayed but increasing caseloads resulting in declining inequality in the distribution towards the end of the epidemic (figures 3*a*,*b* and 4*a*). Our empirical findings from Mumbai, Cape Town and Lagos conform with this modelled outcome, though Rio de Janeiro, Dhaka and Manila do not (figure 1). More surveys and effective ongoing infection surveillance in urban slums of the global South will be required to better understand the true nature and extent of spread in these neighbourhoods, before we draw meaningful conclusions about these discrepancies. It is also possible that the difference in responses to policy measures (such as physical distancing) in slums and non-slums could be yielding varying impacts on the networks of physical contacts in these vastly different settings, and these variations could be pertinent in understanding discrepancies between the model and observation. We discuss the nature of these differences in the Discussion section.

Our model suggests that both in terms of cumulative caseload outcomes at the end of the epidemic (figure 3*a*), as well as (higher and earlier) peak caseloads during the epidemic (figure 4*b*), slum neighbourhoods are much worse off than non-slums in an epidemic. We find that the nature of outcomes described here is robust to a wide range of model parameter choice, such as population of the city system (*N*), probability of transmission (*p*) and number of neighbourhoods (*H*), as well as changes in network structure and mechanism of populating neighbourhoods (detailed results in electronic supplementary material, appendix S3).

In summary, our modelled outcomes are in broad agreement with the empirical observations, suggesting that the nature of these outcomes is more generally reflective of epidemic spread in cities with slums.

## 5. Discussion

Urban slums reflect increased demographic growth, migration, population densities and poverty, which are the main processes found to be linked with prevalence of infectious diseases [36]. There is evidence to suggest that slum populations scale super-linearly with city size [37], meaning that larger cities have more than proportionally larger slums. It is anticipated that there will be over 40 megacities in the world by 2030 and most will be located in the developing world [38]. The evolution of larger slums and higher population densities will mean that slums will continue to be at the forefront of epidemics, both in terms of public health and socioeconomic outcomes.

Our findings suggest that slum populations are among the most vulnerable urban populations in an outbreak. However, as pointed out earlier, there appear to be significant lacunae in our understanding of the true nature of spread within slum neighbourhoods due to a lack of adequate testing in these environments. It is only through limited surveys are we able to estimate the extent of difference in the infection rates between slums and non-slums. Given this high-risk profile of slums, it is imperative that cities develop better disease surveillance and testing strategies for slums, and also a deeper understanding of the effects of factors such as health policy and community on epidemy outcomes in such neighbourhoods [39].

We find that both in terms of peak and cumulative cases, slum neighbourhoods are more vulnerable than non-slum neighbourhoods in cities, given the nature of their physical contact networks. Even as strategies such as physical distancing and lockdown are being adopted to combat the spread of COVID-19, it important to consider the fact that slum populations access and use common public toilets and water sources on a daily basis, meaning that these measures to combat spread become ineffective in the face of basic human needs. Essentially, slum dweller networks cannot exclude these forced, physically proximate daily connections associated with access to such basic needs, and that their networks of physical proximity cannot be reduced to levels feasible for non-slum households. For instance, using Census of India 2011 data, we find that the population densities per communal toilet in the slums of Mumbai, Hyderabad and Pune are 411, 418 and 889, respectively; and the population densities per public water tap or hydrant in the slums of Kolkata, Bengaluru and Jaipur are 94, 112 and 121, respectively. Recent studies on the spread of COVID-19 in Indian cities have reiterated that slum residents have been unable to effectively follow physical distancing measures [7,8,40]. Therefore, long-term solutions to containing epidemic spread in slum environments lies in ensuring that slum settlements are provided with functioning environmental infrastructure for piped running water and private sanitation, waste management and electricity, in addition to basic health infrastructure such as primary healthcare facilities [41–43].

The immediacy of the crisis and its current impacts on slum settlements requires health departments in developing countries to prepare specific guidelines for physical distancing in high-density settlements that are clearly communicated and can be implemented by slum dwellers, so that their exposure risks are minimized [44]. Other immediate measures mooted to protect residents of slum settlements include institution of slum emergency planning committees, guarantee of payments to the poor, implementing strategies for healthcare, mobility, and solid waste collection, and training and deployment of community health workers [45,46].

Despite these constraints in slum neighbourhoods, it is important to point out that community action in conjunction with targeted state intervention has meant that some slum neighbourhoods have been able to effectively counter the spread of infection in the current COVID-19 pandemic. Especially relevant in this case is the case of the Dharavi slum in Mumbai where a sustained programme of community engagement, proactive door-to-door screening in high-risk zones, mobilizing private practitioners and providing basic medical equipment (PPE kits, pulse oximeters, thermal scanners) enabled the local administration to rein in the spread of the virus [9]. While examples such as this offer a potential blueprint for containment in urban slum neighbourhoods, sustained action will be required to ensure that urban slums are better prepared and less vulnerable to future epidemics.

## 6. Conclusion

We study the evolution of the COVID-19 epidemic across neighbourhoods within a city, for a set of metropolises in the developing world. We find an unequal distribution of cases, with a small number of the most densely populated neighbourhoods containing a significant proportion of total caseload across all cities, as illustrated by a$\;k_{f}=0.69$ across these cities. This finding appears to hold across scales, with national case distribution across these states/provinces also displaying similar inequality in case distribution. We also find that neighbourhoods with the highest case densities—both in terms of population and area—contain the largest slums in these cities, and that consequently the urban poor in slums are at the highest risk in this epidemic. Using a simple network model, we simulate the emergence of differential outcomes for slums and non-slums in a city. Model outcomes replicate both unequal distribution of cases as well as higher case densities in high-density neighbourhoods, suggesting that these outcomes are reflective of outbreaks in general for cities with slums. In addition, simulations also predict that as the epidemic progresses, distribution of cases across neighbourhoods becomes less unequal, and that both peak caseloads and cumulative caseloads are worse for slum neighbourhoods vis-à-vis non-slum neighbourhoods in cities.

Given these outcomes, we discuss the need for long-term investments in creating sanitary environments in slums, as well as shorter term measures including community mobilization to control the spread of COVID-19 in dense urban settlements. We also discuss the need for better ongoing data on the spread of infections in urban slums.

## Supplementary Material

Supplementary Information

## Data accessibility

All data are from public sources, outlined in detail in electronic supplementary material, appendix S1.
