# Supplementary material for: Spread of COVID-19 in urban neighbourhoods and slums of the developing world
Source: J R Soc Interface. 2021 Jan 20;18(174):20200599. doi: 10.1098/rsif.2020.0599 (PMC7879756; doi:10.1098/rsif.2020.0599)
Supplement: Supplementary Information [file rsif20200599supp1.pdf]

Spread of COVID-19 in urban neighbourhoods and slums of the developing world

Anand Sahasranaman[1,2,*] and Henrik Jeldtoft Jensen[2,3,#]

[1]Division of Sciences and Division of Social Sciences, Krea University, Sri City, AP 517646, India

[2]Centre for Complexity Science and Dept. of Mathematics, Imperial College London, London SW72AZ, UK.

[3]Institute of Innovative Research, Tokyo Institute of Technology, 4259, Nagatsuta-cho, Yokohama 226-8502, Japan.

[*] Corresponding Author. Email: anand.sahasranaman@krea.edu.in

[#] Email: h.jensen@imperial.ac.uk

SUPPLEMENTARY INFORMATON: Appendices

Appendix 1: Data sources and methods

Data on population, areas, and population densities for sub-city units across the 6 cities was obtained from multiple sources listed below:

1. Mumbai: Population data at the level of Mumbai city's 24 wards is available from the Census of India 2011 at https://www.censusindia.gov.in/2011census/population_enumeration.html. Area and population density data has been collated by volunteer efforts and is available at https://github.com/mickeykedia/Mumbai-Population-Map/blob/master/ward_level_collated.csv.
2. Cape Town: Population, area, and density data for Cape Town's 58 main places - suburbs, towns, and townships - is available from Census of South Africa 2011 data, compiled and visualized at https://census2011.adrianfrith.com/place/199. Detailed data on places is also available from Statistics South Africa at http://www.statssa.gov.za/?page_id=993&id=city-of-cape-town-municipality
3. Rio de Janeiro: Population and density data at the level of Rio's 163 bairros is available from the Brazilian census IBGE https://cidades.ibge.gov.br/brasil/rj/rio-de-janeiro/pesquisa/19/29761, which is also the basis for Wikipedia's pages on Rio's bairros at https://pt.wikipedia.org/wiki/Lista_de_bairros_da_cidade_do_Rio_de_Janeiro.
4. Manila: Population and density data for Manila's 16 districts is obtained from the Philippine Statistics Authority at https://psa.gov.ph/content/population-counts-legislative-district-based-2015-census-population, and Wikipedia's Manila page at https://en.wikipedia.org/wiki/Manila.
5. Lagos: Data on population and density for the 16 Local Government Areas (LGAs) of Lagos are obtained from the Lagos State Government Digest of Statistics 2017, available at: http://lagosstate.gov.ng/Digest_of_Statistics_2017.pdf.
6. Dhaka: Population and density of the 41 thanas of Dhaka are obtained from the Bangladesh Population and Housing Census 2011 available at

https://web.archive.org/web/20151208044832/http://www.bbs.gov.bd/WebTestApplication/userfiles/Image/National%20Reports/Union%20Statistics.pdf, and also Banglapedia - the national encyclopaedia of Bangladesh by scholars at http://en.banglapedia.org/index.php?title=Main_Page.

Data on Covid-19 caseloads across states, provinces, or districts of the 6 nations was obtained from

1. India: https://api.covid19india.org/csv/latest/state_wise.csv
2. South Africa: https://covid19data.co.za/provinces/
3. Brazil: https://en.wikipedia.org/wiki/Template:COVID-19_pandemic_data/Brazil_medical_cases
4. Bangladesh: https://www.iedcr.gov.bd/
5. Nigeria: https://covid19.ncdc.gov.ng/

Data on Covid-19 caseloads for different cities were obtained from the following sources:

1. Mumbai: The Brihanmumbai Municipal Corporation's (BMC) daily coronavirus update at the ward level is available at http://stopcoronavirus.mcgm.gov.in/key-updates-trends
2. Cape Town: The Western Cape government has a Covid-19 response site at https://coronavirus.westerncape.gov.za/covid-19-dashboard, but this does not provide regular updates and details at the town and suburb level. Data at that level is released as an occasional report. We use the report provided for June 1 available at https://coronavirus.westerncape.gov.za/files/atoms/files/WC%20Suburb%20and%20town%20cases%20-%201%20June.pdf
3. Rio de Janeiro: Data for Covid-19 at the level of Rio de Janeiro's bairros is available at Data Rio https://experience.arcgis.com/experience/38efc69787a346959c931568bd9e2cc4
4. Manila: The City of Manila has an irregularly updated Covid-19 situation report at the level of the city's districts available at http://manila.gov.ph/2020/06/covid-19-monitoring/.
5. Lagos: The Nigeria Centre for Disease Control releases daily situation reports capturing the spread of Covid-19 across states in Nigeria at https://ncdc.gov.ng/diseases/sitreps/?cat=14&name=An%20update%20of%20COVID-19%20outbreak%20in%20Nigeria. The only data available on the spread with the Local Government Areas (LGAs) of Lagos was released by the Lagos State Commissioner for Health, Professor Akin Abayomi and reported in the media at https://www.vanguardngr.com/2020/05/covid-19-lagos-lgas-and-number-of-confirmed-cases/ and https://dailypost.ng/2020/05/09/lagos-releases-lga-with-highest-covid-19-cases-see-full-list/.
6. Dhaka: Covid-19 data at the level of Dhaka's thanas is available at The Bengal Institute's Covid-19 resource site https://bengal.institute/research/covid19dhaka/.

Appendix 2: Mapping slums to neighbourhoods

We identified slums and then mapped them on to respective neighbourhoods across the 6 cities under consideration. We describe this process for each city.

Mumbai: We used a Mumbai slums map, which was part of the Municipal Corporation of Greater Mumbai's Mumbai City Development Plan 2005-2025, available at https://www.dur.ac.uk/geography/everyday_sanitation/the_research_project/ to allot slums to neighbourhoods. Based on a visual inspection of the spread of slums across wards in Mumbai, we assign 11 wards as 'neighbourhoods with slums': G-North (containing the Dharavi slum), G-South, F-South, L, N, H-East, M-East, M-West, K-East, K-West, and P-North.

Cape Town: We use settlement-level information from Statistics South Africa for places in Cape Town at http://www.statssa.gov.za/?page_id=993&id=city-of-cape-town-municipality as well as Wikipedia listings of South Africa's largest townships (slums) at https://en.wikipedia.org/wiki/Township_(South_Africa) to construct a list of 8 settlements, which we classify as neighbourhoods with slums in Cape Town: Khayelitsha, Mitchells Plain, Gugulethu, Delft, Philippi, Nyanga, Langa, and Mfuleni.

Rio de Janeiro: We use census information from IBGE at https://cidades.ibge.gov.br/brasil/rj/rio-de-janeiro/pesquisa/19/29761 and Wikipedia's listing of Rio de Janeiro's favelas at https://pt.wikipedia.org/wiki/Lista_de_favelas_da_cidade_do_Rio_de_Janeiro to construct a list of 41 bairros that we label as neighbourhoods with slums: Rocinha, Jacarezihno, Mare, Cidade de Deus, Complexo do Alemao, Mangueira, Penha, Acari, Tijuca, Costa Barros, Ramos, Benfica, Pavuna, Encantado, Lins de Vasconcelos, Manguinhos, Madureira, Inhaumos, Rio Comprido, Iraja, Anchieta, Vigario Geral, Guadalupe, Cordovil, Piedade, Jacare, Parada de Lucas, Copacabana, Tomas Coelho, Magalhaes Bastos, Realengo, Bangu, Jacarepagua, Andarai, Bras de Pina, Honorio Gurgel, Engenho Novo, Turiacu, Padre Miguel, Coelho Neto, and Engenho de Dentro.

Dhaka: We use information from the Slums of Urban Bangladesh, Mapping and Census 2005 report produced by the Centre for Urban Studies (synopsis of main results available at https://www.thedailystar.net/news-detail-93293) and identify a list of 12 thanas as neighbourhoods with slums: Mirpur, Gulshan (containing the Korail slum), Mohammadpur, Jatrabari, Lalbagh, Sutrapur, Chak Bazar, Gendaria, Hazaribagh, Kotwali, Kamrangir Char, and Shyampur.

Lagos: We use information from news reports (https://nationaldailyng.com/nigeria-tops-list-of-worst-slums-in-africa/, https://www.bbc.com/news/av/world-africa-45736981/lagos-floating-slum-makoko-what-s-it-like-to-live-there, https://www.vanguardngr.com/2019/07/lagos-megacity-in-dire-need-of-improved-sanitation/) and Wikipedia's list of slums in Nigeria (Lagos) at https://en.wikipedia.org/wiki/List_of_slums_in_Nigeria to identify 5 Local Government Areas (LGAs) as neighbourhoods with slums: Agege, Ajeromi-Ifelodun, Mushin, Somolu, Lagos Island, and Lagos Mainland (containing the floating Makoko slum).

Manila: We use a report on the slums of Metro Manila (https://www.ucl.ac.uk/dpu-projects/Global_Report/cities/manila.htm) and Wikipedia's listing of slums in Metro Manila (https://en.wikipedia.org/wiki/Slums_in_Metro_Manila#cite_note-1) to identify two districts in the City of Manila as neighbourhoods with slums: Tondo and San Andres.

Appendix 3: Sensitivity analysis and robustness checks

Table A3.1 presents the parameter values and initial conditions for simulations testing sensitivity to population size, neighbourhood count, and probability of transmission. Across the set of parameter variations presented here, we find that the outcomes of the model to be robust.

SENSITIVITY TO	Population	Neighbourhood count	Transmission probability
**Parameters**			
Population – number of network nodes, N	$\mathbf{1,000}$ &$\mathbf{100,000}$	10,000	10,000
Number of edges from new node to extant nodes, m (BA network)	50	50	50
Number of neighbourhoods, H	20	$\mathbf{10}$ & $\mathbf{40}$	20
Probability of node to node transmission, p	0.004	0.004	$\mathbf{0.002}$ & $\mathbf{0.006}$
Number of iterations in one simulation of model, T_f	120	120	120
Number of simulations	100	100	100
**Initial Conditions**			
Number of susceptible nodes, $S(0)$	9,999	9,999	9,999
Number of infectious nodes, $I(0)$	1	1	1
Number of recovered nodes, $R(0)$	0	0	0

Table A3.1: *Parameter and initial conditions for sensitivity analysis*

In addition to these sets of simulations, we also test model sensitivity to:

i. Changing the network type for the dynamics: Instead of the Barabasi-Albert network, we model dynamics on an Erdos-Reyni random graph. All parameters for this simulation are as in base case, except that the link probability parameter to generated edges in the graph is $p_{link} = 0.005$. Neighbourhood allocation for agents also follows the same algorithm as in base case.

ii. Changing the algorithm for neighbourhood allocation: On the Barabasi-Albert network, instead of ordering agents by degree of node and then allotting then in blocks of N/H to each neighbourhood as in the base case, we now try three variations:
   a. allot them in blocks of $N/2H$ agents and repeat the process twice, such that all neighbourhoods have N/H agents at the end of the allocation process.
   b. allot them in blocks of $N/4H$ agents and repeat the process four times, such that all neighbourhoods have N/H agents at the end of the allocation process.
   c. allot them in blocks of $N/10H$ agents and repeat the process ten times, such that all neighbourhoods have N/H agents at the end of the allocation process.

Population size: We vary population size ($N = 1000$ and $N = 100{,}000$) and find that both the outcomes for distribution of cases across neighbourhoods and the evolution of cumulative caseloads across neighbourhoods of varying average density show behaviour that is similar to the base case (Fig. A3.1). The higher the density of a neighbourhood, the earlier and steeper its rise in case density. We also see that the inequality in distribution of cases across neighbourhoods decreases over time as in the base case. We also find that, while nature of outcomes is consistent across all scenarios, both cumulative caseloads and the extent of differential impacts between higher and lower density neighbourhoods increase with decreasing population.

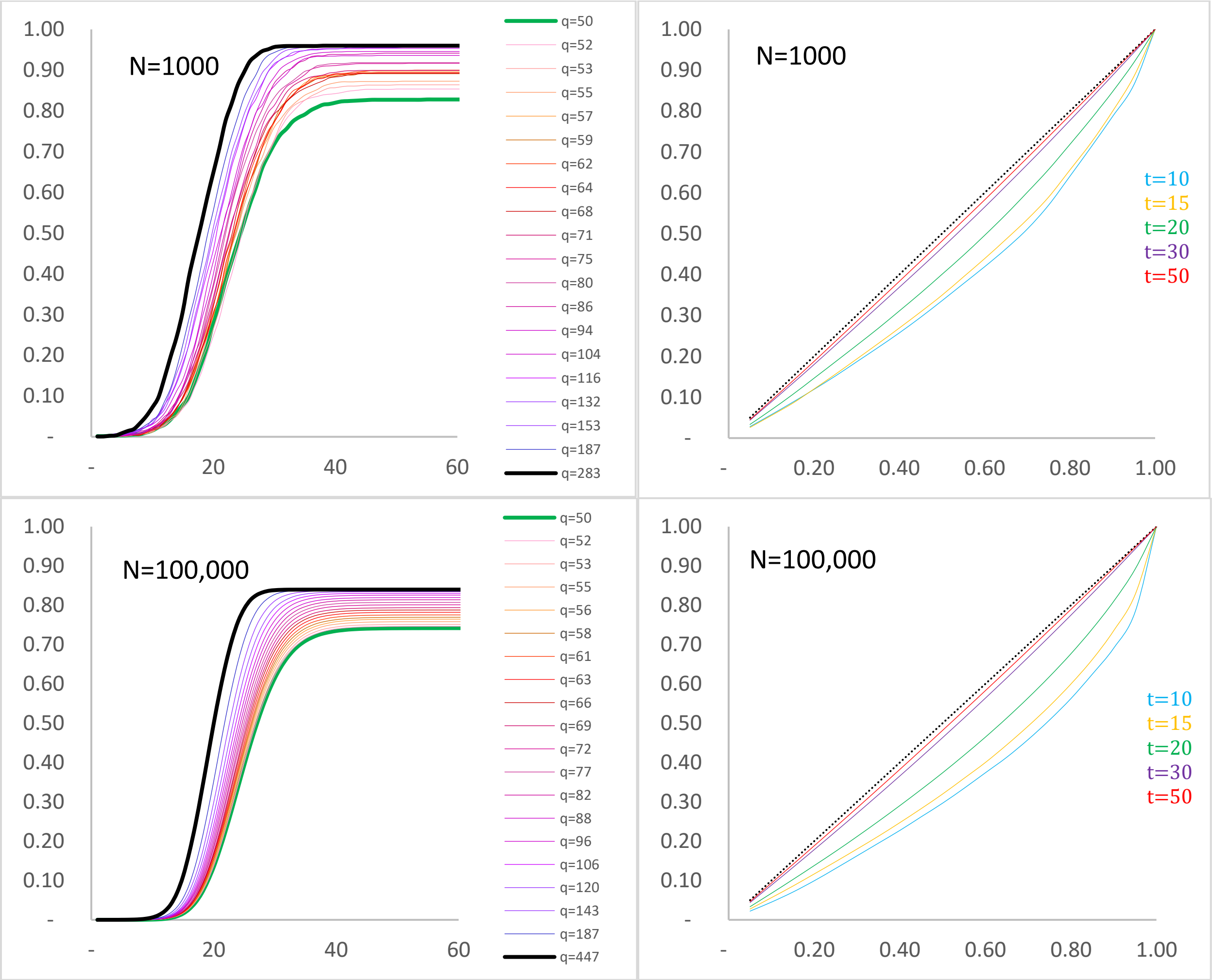

Figure A3.1: *Simulation outcomes for $N = 1000$ and $N = 100{,}000$.* Top left and right show the evolution of cumulative caseloads and distribution of cases across neighbourhoods respectively for $N = 1000$, and the bottom left and right show the same for $N = 100{,}000$.

Neighbourhood count: We vary neighbourhood count ($H = 10$ and $H = 40$) and find that both the outcomes for distribution of cases across neighbourhoods and the evolution of cumulative caseloads across neighbourhoods of varying average density show behaviour that is in concordance with the base case (Fig. A3.2). The higher the density of a neighbourhood, the

earlier and steeper its rise in case density. We also see that the inequality in distribution of cases across neighbourhoods decreases over time as in the base case.

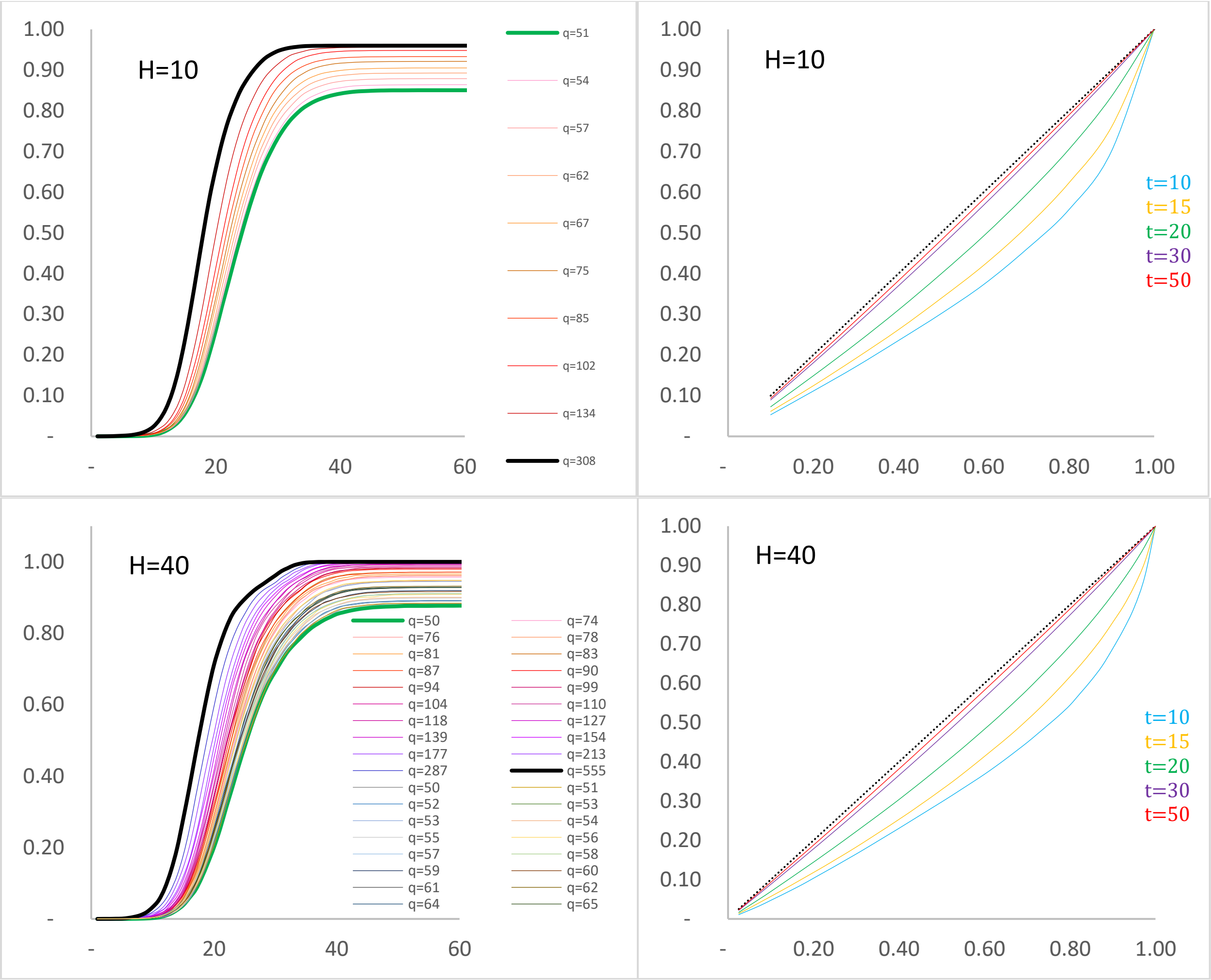

Figure A3.2: *Simulation outcomes for $H = 10$ and $H = 40$.* Top left and right show the evolution of cumulative caseloads and distribution of cases across neighbourhoods respectively for $H = 10$, and the bottom left and right show the same for $H = 40$.

Transmission probability: We vary transmission probability ($p = 0.002$ and $p = 0.006$) and find that both the outcomes for distribution of cases across neighbourhoods and the evolution of cumulative caseloads across neighbourhoods of varying average density show behaviour that is in concordance with the base case (Fig. A3.3). The higher the density of a neighbourhood, the earlier and steeper its rise in case density. We also see that the inequality in distribution of cases across neighbourhoods decreases over time as in the base case. For $p = 0.002$, we find that the time taken for the differential dynamics to emerge is longer, while it is much shorter for $p = 0.006$ – and, the nature of differential impacts remains the same, the extent of difference between the higher and lower density neighbourhoods is exacerbated for lower p.

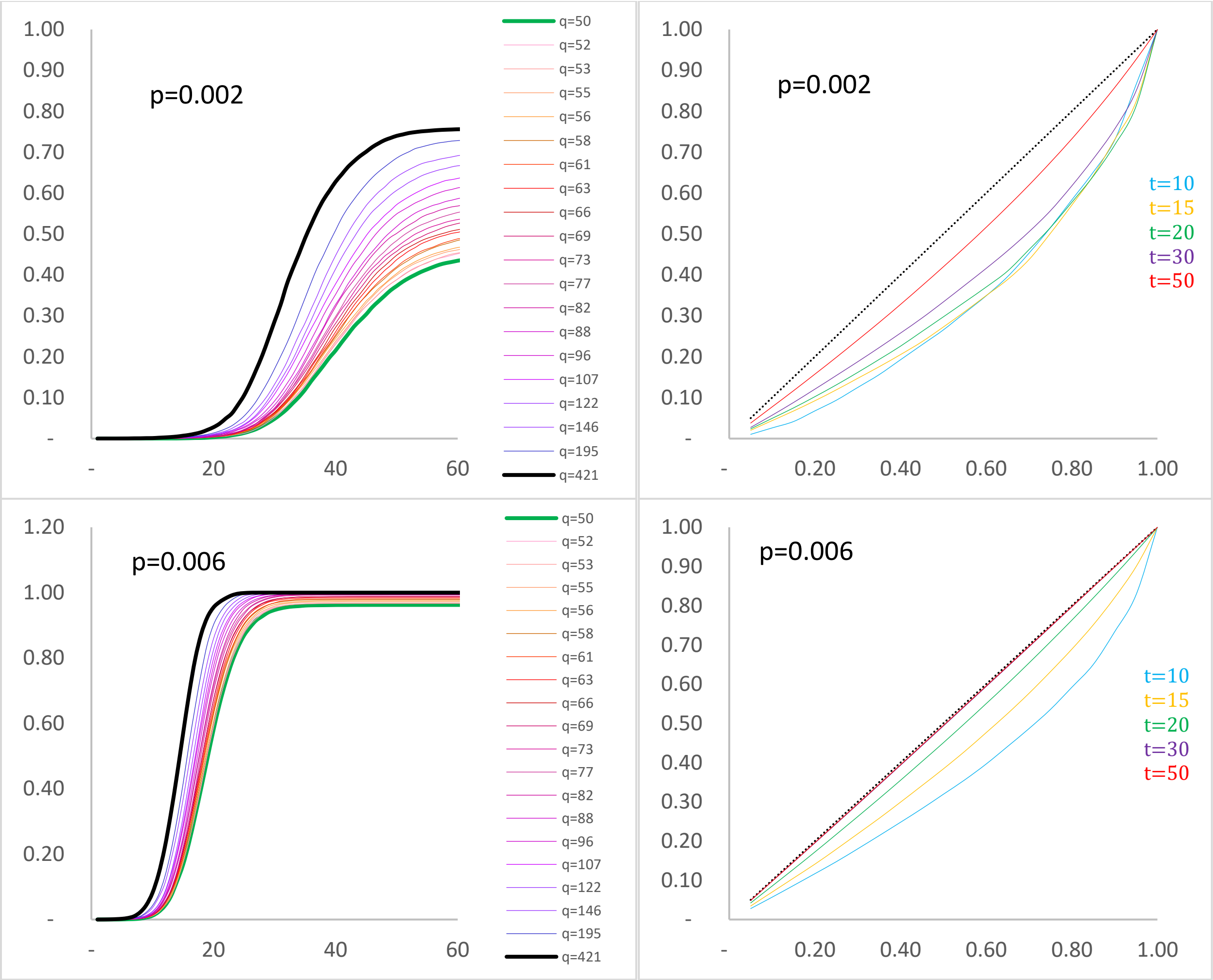

Figure A3.3: *Simulation outcomes for* $p = 0.002$ *and* $p = 0.006$. Top left and right show the evolution of cumulative caseloads and distribution of cases across neighbourhoods respectively for $p = 0.002$, and the bottom left and right show the same for $p = 0.006$.

Network type: We vary network type and simulate dynamics on an Erdos-Reyni random graph and find that both the outcomes for distribution of cases across neighbourhoods and the evolution of cumulative caseloads across neighbourhoods of varying average density show behaviour that is in concordance with the base case (Fig. A3.4). The higher the density of a neighbourhood, the earlier and steeper its rise in case density, though cumulative caseloads are, on average, lower in this scenario than the base case. We also see that while inequality in distribution of cases across neighbourhoods decreases over time, the extent of inequality in the distribution is much lesser (even initially) than in the case of the Barabasi-Albert graph – this is a function of the randomness in the process of edge creation in this network, unlike the preferential attachment in operation in the Barabasi-Albert graph. Given real distribution data for the six cities in our analysis, this suggests that the true nature of networks of physical proximity in cities of the developing world is closer to the Barabasi-Albert network rather than a random network like the Erdos-Reyni.

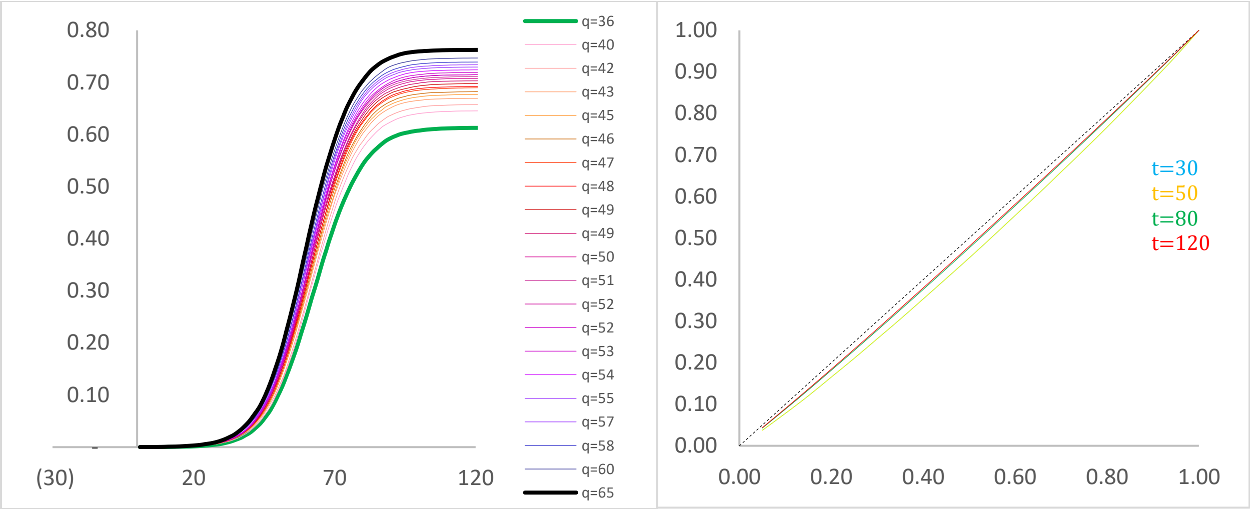

Figure A3.4: *Simulation outcomes for Erdos-Reyni graph.* Left and right show the evolution of cumulative caseloads and distribution of cases across neighbourhoods respectively.

Algorithm to populate neighbourhoods: We vary algorithm to populate neighbourhoods as indicated earlier and find that both the outcomes for distribution of cases across neighbourhoods and the evolution of cumulative caseloads across neighbourhoods of varying average density show behaviour that is in concordance with the base case (Fig. A3.5). The higher the density of a neighbourhood, the earlier and steeper its rise in case density. However, across the three scenarios discussed here, we find that as the range of population densities across neighbourhoods decreases, the differential impacts between the densest and sparsest neighbourhoods decrease, as we would expect. We also see that the inequality in distribution of cases across neighbourhoods decreases over time as in the base case, though the initial inequality decreases as the range of population densities across neighbourhoods decreases.

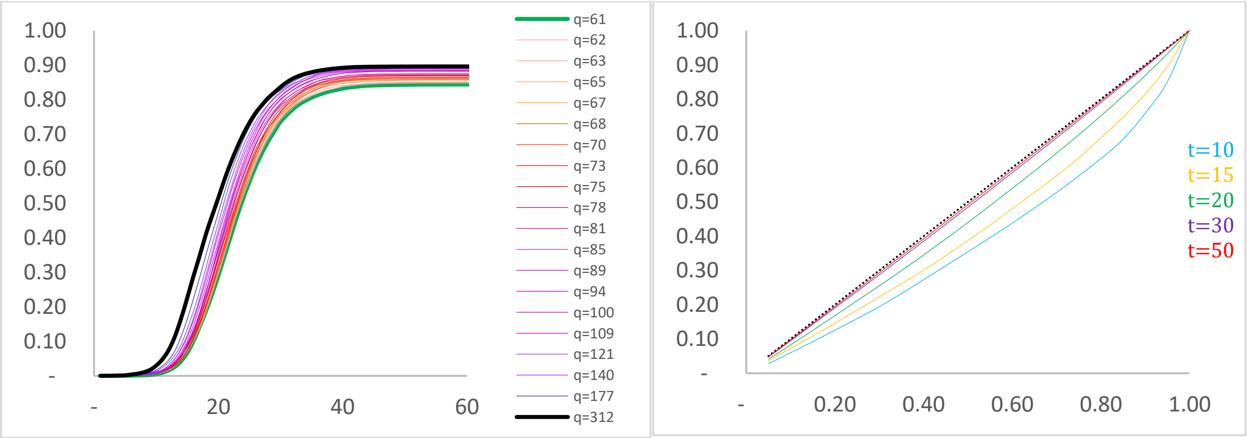

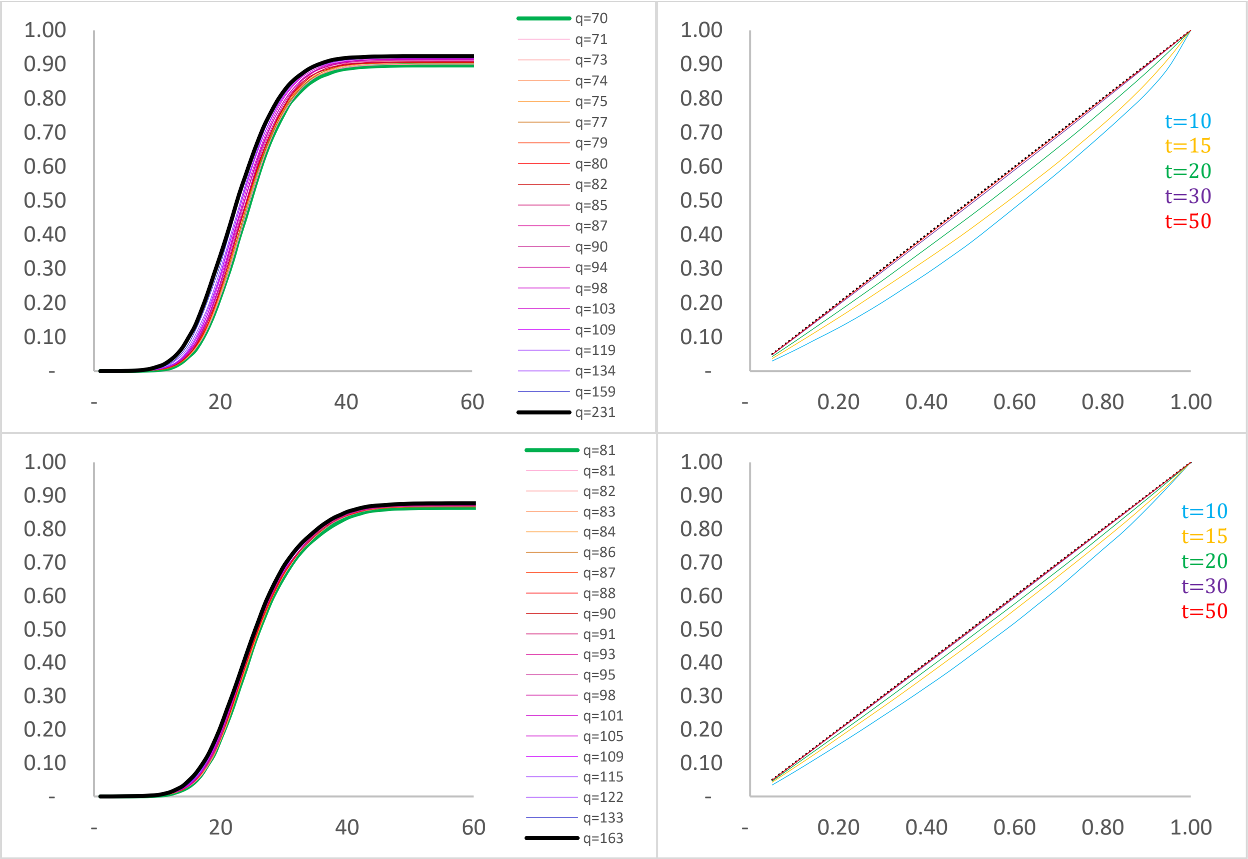

Figure A3.5: *Simulation outcomes for changes in neighbourhood composition.* Top left and right show the evolution of cumulative caseloads and distribution of cases across neighbourhoods respectively for $N/2H$ agents populated at a time in a neighbourhood and repeated twice. Middle left and right show the outcomes for $N/4H$ agents populated at a time in a neighbourhood and repeated four times. Bottom left and right show outcomes for $N/10H$ agents populated at a time in a neighbourhood and repeated ten times.
